# Adaptation of emergency surgical care during successive COVID-19 waves: a single-center analysis of surgically treated acute appendicitis

**DOI:** 10.1186/s12893-026-03754-x

**Published:** 2026-04-23

**Authors:** Michael Hoffmann, Matthias Schrempf, Luis Arbona de Gracia, Anna Muzalyova, Marie Freitag, Christoph Römmele, Florian Sommer, Matthias Anthuber, Sebastian Wolf

**Affiliations:** 1 Klinik für Allgemein, Viszeral und Transplantationschirurgie, Universitätsklinikum Augsburg, Stenglinstraße 2, Augsburg, 86156 Deutschland; 2 Department of Anaesthesiology, Ludwig-Maximilians-University Hospital Munich, Munich, Germany; 3 Institute for Digital Medicine, University Hospital Augsburg, Augsburg, Germany; 4 Department of Gastroenterology, University Hospital Augsburg, Augsburg, Germany; 5 Department of General, Visceral, Thoracic and Minimally-Invasive Surgery, City Hospital Bad Tölz, Bad Tölz, Germany

**Keywords:** COVID-19, SARS-CoV-2 infections, Appendicitis, Pandemic

## Abstract

**Background:**

During the first wave of the COVID-19 pandemic, several studies – including our own - demonstrated a significant decrease in emergency surgical presentations, including acute appendicitis (AA). While these early findings are well documented, little is known about how presentation patterns and clinical outcomes evolved across subsequent pandemic waves. This study examines longer-term trends in AA care across three distinct pandemic phases, highlighting how healthcare systems and patient responses adapted over time within a tertiary surgical setting.

**Methods:**

We conducted a retrospective single-center cohort study. Patients undergoing appendectomy between March 2020 and December 2021 were assigned to distinct pandemic phases based on national SARS-CoV-2 incidence (wave 2, wave 3, and wave 4). These were each compared separately to a pre-pandemic reference cohort treated between January 2019 and February 2020. Demographic data, clinical presentation, laboratory values, histological findings, and postoperative outcomes were analyzed.

**Results:**

Throughout the pandemic period, the number of appendectomies remained lower than in the pre-pandemic reference group, particularly during high-incidence phases. Patients in wave 2 presented with more advanced disease, including significantly higher rates of perforation and peritonitis. In waves 3 and 4, disease severity and postoperative outcomes were comparable to the pre-pandemic reference period.

**Conclusion:**

This long-term analysis suggests that the initial shift toward more advanced disease among patients undergoing appendectomy was not sustained, with presentation patterns and outcomes tending toward pre-pandemic levels during later pandemic waves. These findings may reflect adaptive processes within the healthcare system, despite ongoing external stress.

## Introduction

Since its outbreak in December 2019, SARS-CoV-2 infections showed exponential growth and worldwide spread, leading to a global pandemic. This affected healthcare systems resulting in a shortage of critical care capacity and healthcare workers due to the need for COVID-19 treatment. Thus, elective and urgent operations had to be postponed to preserve resources. In many institutions worldwide, the number of admissions to emergency departments fell significantly, especially at the beginning of the pandemic [1–5].

Accounting for a considerable proportion of emergency surgical admissions, patients with acute appendicitis (AA) represent one of the most common admissions to the emergency department requiring surgical treatment [6]. Around 135,000 patients undergo appendectomy in Germany every year [7]. Therefore, AA provides a useful model for examining changes in emergency surgical care under conditions of system-wide stress.

A large number of studies and meta-analyses showed that fewer patients presented and were treated with AA at the beginning of the COVID-19 pandemic and the first lockdown [3, 4, 8–10]. However, many groups reported a higher proportion of complicated appendicitis [11–14].

This phenomenon is not fully understood, and there is an ongoing discussion about the reasons [15, 16]. Pandemic-related restrictions during the lockdown and pandemic-related concerns may have contributed to patients deciding to seek medical attention later than usual. Thus, a prehospital delay may have contributed to more advanced disease. In addition, the increased proportion of complicated appendicitis may not necessarily reflect a rise in absolute numbers of severe cases, but could result from a decrease in uncomplicated cases. Some patients might have been successfully treated as an outpatient with antibiotics which may have reduced the number of patients with uncomplicated appendicitis treated as inpatients [17]. Finally, recent evidence suggests that an increase in spontaneously resolving uncomplicated appendicitis might be an important explanation for the higher proportion of complicated appendicitis [15].

Although SARS-CoV-2 infections have continued to occur, the overall impact on healthcare systems has evolved over time. Medical institutions adapted to prolonged pandemic conditions through organizational changes, modified workflows, and improved infection control measures. While most clinical studies have focused on the early phase of the pandemic, an increasing number of reports now address longer-term effects on healthcare delivery.

The aim of the present study was to analyze longer-term patterns of presentation and outcomes among patients undergoing appendectomy during successive COVID-19 waves. Using acute appendicitis as a frequent and well-defined emergency surgical condition, we compared distinct pandemic phases with a pre-pandemic reference period to assess temporal changes in surgically treated AA within a tertiary referral center.

## Materials and methods

This is a single-center retrospective cohort analysis undertaken at the high-volume university hospital Augsburg, Germany. This study builds upon our previous work, which analyzed wave 1 of the COVID-19 pandemic (March–April 2020), including incidence and severity of acute appendicitis (AA) during that initial phase [14]. The current study extends this analysis to subsequent pandemic phases in order to assess long-term patterns of presentation and outcomes.

Approval for this study was granted from the ethics committee of the Ludwig-Maximilians-University Munich (the ethic number 22–0661). The requirement for informed consent was waived in the light of the retrospective and anonymous nature of the study.

ICD-10 diagnostic code K35 for acute appendicitis and operation codes 5-470, 5-471 and 5-479 for appendectomy were used to detect patients with AA. All consecutive patients between January 1 2019 and December 31 2021 aged 16 years or older were identified from the institutional electronic database.

Patients were divided into two main groups:

- Group A: pre-pandemic (January 1, 2019 – February 28, 2020).
- Group B: pandemic (March 1, 2020 – December 31, 2021).

To assess the long-term impact of the pandemic, Group B was further stratified into three periods based on local epidemiological data on SARS-CoV-2 incidence in Bavaria and at our institution:

- Wave 2: October 1 – December 31, 2020.
- Wave 3: March 1 – May 31, 2021.
- Wave 4: September 1 – November 30, 2021.

**Fig. 1:**
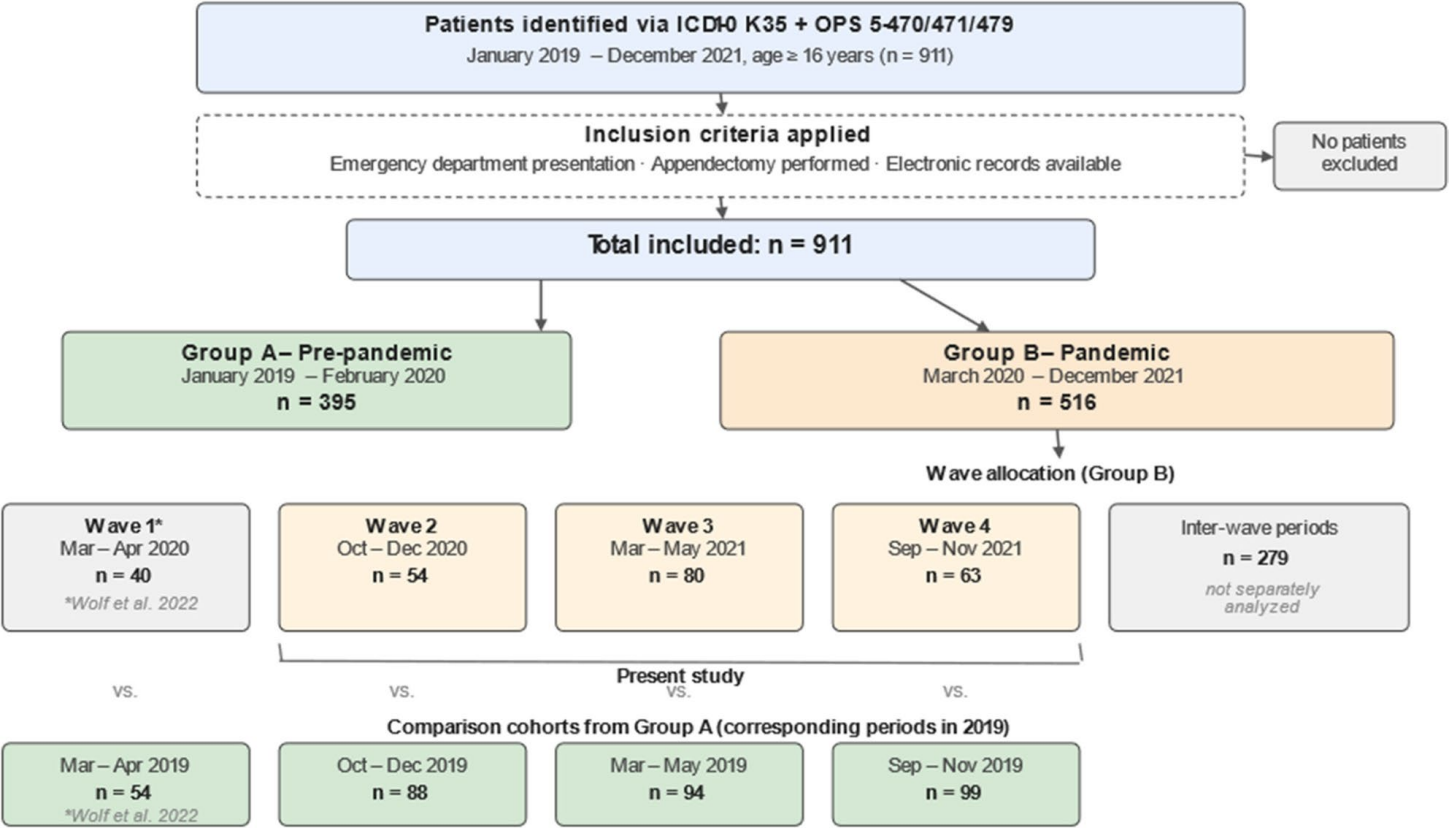
Patient identification and allocation flow diagram. * Wave 1 was analyzed in a prior publication [14] and is shown for completeness; dashed lines indicate groups not analyzed in the present study

The identification and allocation process is illustrated in Fig. 1.

Wave 1 (March – April 2020) was analyzed in our prior publication [14] and is referenced here for contextual purposes only.

**Fig. 2:**
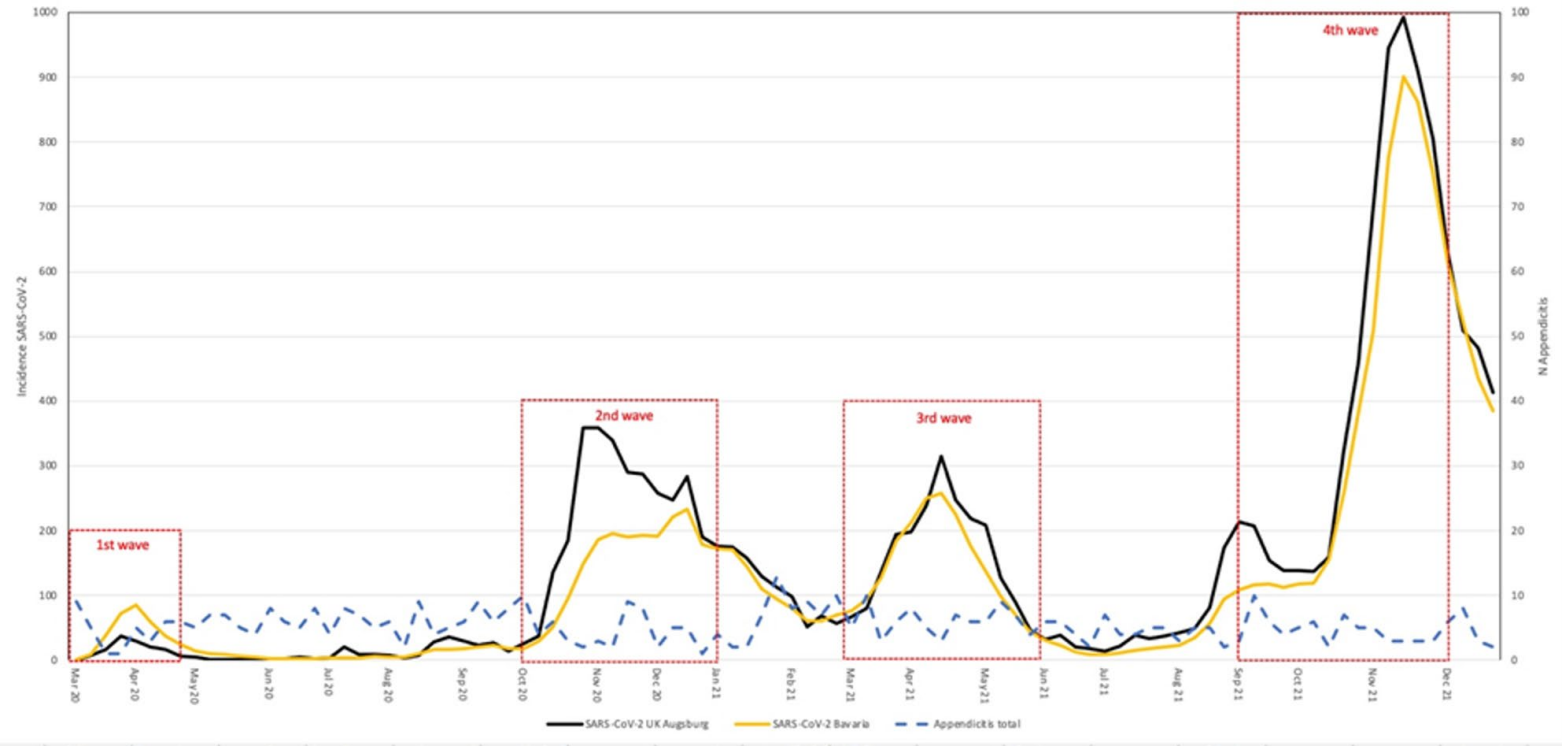
Incidence of SARS-CoV2 and number of patients with acute appendicitis

Pandemic waves were defined based on sustained periods of elevated SARS-CoV-2 incidence in Bavaria, as reported by the Robert Koch Institute (RKI), and corroborated by institutional admission data for SARS-CoV-2-positive patients at our center (Fig. 2). The wave periods correspond to the nationally recognized pandemic waves in Germany: wave 2 (October–December 2020), wave 3 (March–May 2021), and wave 4 (September–November 2021). No single numerical threshold was applied; rather, the wave boundaries were chosen to capture the peak incidence periods as visualized in Fig. 2.

In addition to AA incidence and clinical outcomes, we evaluated regional trends in SARS-CoV-2 infections using case numbers reported for Bavaria and for our hospital. This was intended to contextualize the timing and extent of pandemic waves during the study period and to interpret patient presentation patterns relative to local public health conditions. All patients admitted to hospital with PCR-verified SARS-CoV-2 infection were counted and set into context with all verified SARS-CoV-2-infected patients in Bavaria. Patients included in this study were those who underwent surgery for suspected acute appendicitis, as assessed by the attending surgeon. The diagnosis was based on typical clinical presentation, including abdominal pain, leukocytosis, and elevated C-reactive protein (CRP). All patients underwent imaging studies, at minimum an abdominal ultrasound, in accordance with institutional protocols. Patients in whom the appendix appeared macroscopically normal during surgery were still included, as the clinical decision to operate had been based on findings suggestive of acute appendicitis (negative appendectomy).

Demographics of patients were evaluated including age, sex, duration of symptoms, leukocyte count and C-reactive protein (CRP). Furthermore, operation technique and peri- and postoperative parameters such as length of hospital stay, macroscopic grade of appendicitis and peritonitis, histologic grading, and postoperative need for antibiotic treatment were analyzed. Disease severity was assessed at two levels: (1) the intraoperative macroscopic grade of appendicitis, as documented by the operating surgeon (categorized as normal, acute, or perforated/abscess), and (2) the histological result from pathological examination of the resected specimen (categorized as no appendicitis, mild/phlegmonous, or severe/necrotic/perforated). The decision to administer postoperative antibiotics was based on intraoperative findings (e.g., presence of peritonitis, perforation, or abscess) and was at the discretion of the attending surgeon. There was no formal institutional protocol mandating antibiotic use for specific intraoperative findings during the study period.

All patients presenting with acute appendicitis at the emergency department were initially PCR-tested for SARS-CoV-2 upon admission. To identify possible nosocomial infections, patients were re-tested twice weekly or if they developed symptoms (e.g., fever, respiratory complaints). Nosocomial infections were defined as SARS-CoV-2 infections diagnosed after hospital admission that were not present at the time of admission.

All statistical analyses were performed using SPSS, version 21 (IBM SPSS Statistics for Windows, version 21.0). Continuous variables were compared using t-test or Wilcoxon rank-sum test depending on the distribution and are shown as mean ± standard deviation. Categorical variables are reported as number and percentage and compared using Fisher’s exact or Chi squared test. A two-sided *p*-value of < 0.05 was considered significant. Missing data were minimal: duration of symptoms was missing in 4 patients, length of stay in 1 patient, and postoperative antibiotic use in 1 patient; all other variables included in Table 2 were complete. Analyses were performed on available cases for the respective variables.

The statistical analyses in this study are exploratory in nature. Given the limited sample sizes within individual waves and the hypothesis-generating purpose of the wave-based comparisons, no correction for multiple testing was applied, as such corrections would be overly conservative in this context. The *p*-values presented should therefore be regarded as descriptive rather than confirmatory. Due to the retrospective design and the variety of outcomes assessed, statistically significant differences should be interpreted with caution, and definitive causal conclusions cannot be drawn from these findings.

## Results

### Clinical characteristics of patients

A total of 395 patients with AA were identified in the period before the pandemic (January 2019 – February 2020) and included as Group A. 516 patients were included in Group B (March 2020 – December 2021). The clinical characteristics of both groups were largely similar, with the exception of a slightly higher age in the pandemic group (Table 1).

**Table 1:** Clinical characteristics of all patients

	Group A (pre-COVID) *n* = 395	Group B (COVID) *n* = 516	*p* value
Age	36.92 ± 16.55	39.64 ± 17.58	0.02
Sex	Male: 202	Male: 280	0.35
	Female: 193	Female: 236	
Duration of symptoms (days)	2.04 ± 2.15	2.24 ± 3.32	0.30
Total leukocyte count (G/l)	12.65 ± 4.58	12.87 ± 4.79	0.48
CRP (mg/dl)	5.44 ± 6.66	5.74 ± 6.12	0.49
Length of stay (days)	3.63 ± 2.51	3.55 ± 2.82	0.66
Operative technique			
0: laparoscopic	374 (94.68%)	482 (93.41%)	0.43
1: open	5 (1.27%)	5 (0.97%)	
2: conversion	12 (3.04%)	24 (4.65%)	
Postoperative Antibiotics	107 (27.1%)	169 (32.75%)	0.07
Peritonitis	105 (26.58%)	147 (28.49%)	0.52
Grade of Appendicitis			
0: normal	26 (6.58%)	29 (5.62%)	0.60
1: acute	285 (72.15%)	362 (70.16%)	
2: perforated/abscess	81 (20.51%)	118 (22.87%)	
Histologic result			
0: no appendicitis	39 (9.87%)	35 (6.78%)	0.28
1: mild/phlegmonous	217 (54.94%)	289 (56%)	
2: severe/necrotic/perforated	135 (34.18%)	176 (34.11%)	
SARS-CoV-2 Infection	0 (0%)	5 (0.97%)	0.07

Continuous variables are presented as mean ± SD and compared using Welch’s t-test. Categorical variables are reported as *n* (%) and compared using Chi² test or Fisher’s exact test (SARS-CoV-2 infection). For categorical variables with three categories (operative technique, grade of appendicitis, histologic result), a single global Chi² test (df = 2) was applied

### Correlation to regional pandemic situation

To provide broader context, we correlated the patients admitted with AA with the regional pandemic situation.

The incidence of SARS-CoV-2 in Bavaria and our hospital, as well as the number of patients with AA, are shown in Fig. 2. For the current analysis, the long-term pandemic period was defined as waves 2–4:

- Wave 2: October 1 – December 31, 2020.
- Wave 3: March 1 – May 31, 2021.
- Wave 4: September 1 – November 30, 2021.

**Fig. 3:**
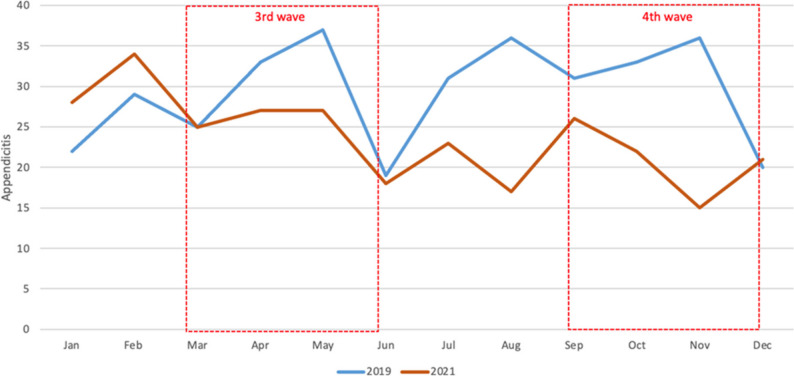
Number of patients with acute appendicitis during 1st and 2nd wave compared to 2019

**Fig. 4:**
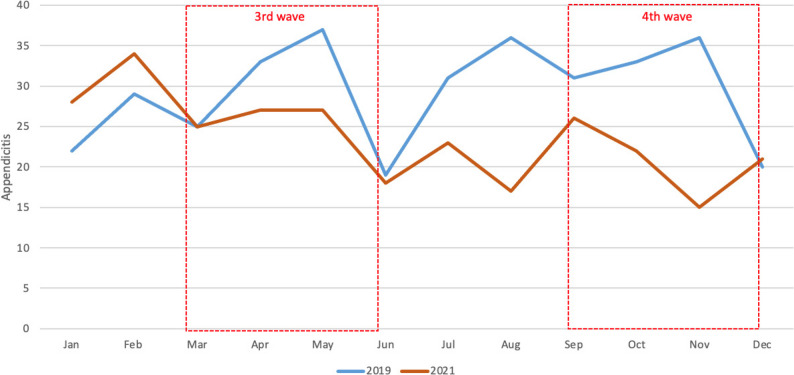
Number of patients with acute appendicitis during 3rd and 4th wave compared to 2019

Figures 3 and 4 show the number of AA during the four waves in comparison to the corresponding timeframe 2019. During wave 1 (March–April 2020), 40 patients underwent appendectomy compared to 54 patients in the corresponding period in 2019, representing a 26% decrease^14^. Patients during wave 1 presented with significantly more advanced disease, including higher rates of perforation and elevated inflammatory markers, consistent with the pattern subsequently observed during wave 2.

The period of wave 2 included 54 patients (group B) in comparison to 88 patients (comparable group A, 2019). This corresponds to a decrease of 38.6% of patients with AA in the ^2n^d pandemic wave. There were 80 patients with AA included in the ^3r^d wave vs. 94 patients in group A (decrease of 14.9%) and 63 patients in wave 4 vs. 99 patients (decrease of 36.4%).

### Demographics and outcome parameter – in correlation to pandemic waves

#### Wave 2

The period from October 1 to December 31, 2020 was defined as the 2nd pandemic wave. There were 54 patients with AA identified in this period compared to 88 patients in the same period in 2019. Baseline characteristics and outcomes are shown in Table 2. Patients in the pandemic group were significantly older (45.85 vs. 34.81 years, *p* < 0.001). Inflammatory markers and length of hospital stay were numerically higher in the pandemic group, without reaching statistical significance individually (CRP: 6.02 vs. 3.90 mg/dl, *p* = 0.069; leukocyte count: 13.65 vs. 12.27 G/l, *p* = 0.073; length of stay: 3.74 vs. 3.28 days, *p* = 0.194). In contrast, surgical and intraoperative indicators of disease severity showed clear differences: patients during wave 2 had significantly more perforations and abscesses (37.0% vs. 11.4%, *p* < 0.001), a higher rate of peritonitis (42.6% vs. 23.9%, *p* = 0.031), more frequent postoperative antibiotic use (42.6% vs. 23.9%, *p* = 0.031), and a higher rate of conversion to open surgery (9.3% vs. 1.1%, *p* = 0.03). Taken together, these findings suggest a more advanced disease presentation during wave 2.

**Table 2:** Baseline characteristics and outcome during wave 2, 3 and 4 compared to corresponding periods in 2019

	Oct 1st to Dec 31st 2019 (*n* = 88)	Oct 1st to Dec 31st 2020 (*n* = 54)	*p* value	Effect (95% CI)	Mar 1st to May 31st 2019 (*n* = 94)	Mar 1st to May 31st 2021 (*n* = 80)	*p* value	Effect (95% CI)	Sep 1st to Nov 30th 2019 (*n* = 99)	Sep 1st to Nov 30th 2021 (*n* = 63)	*p* value	Effect (95% CI)
Age	34.81 ± 15.83	45.85 ± 19.95	< 0.001	+ 11.05 [4.70 to 17.39]	39.07 ± 18.21	42.26 ± 20.04	0.277	+ 3.19 [-2.58 to 8.96]	36.41 ± 17.55	36.73 ± 20.38	0.919	+0.32 [-5.85 to 6.48]
Duration of symptoms (days)	2.11 ± 4.44	2.23 ± 2.25	0.837	+ 0.12 [-1.00 to 1.24]	1.78 ± 1.44	2.05 ± 1.95	0.32	+ 0.27 [-0.26 to 0.79]	2.30 ± 4.32	2.73 ± 2.62	0.435	+ 0.43 [-0.65 to 1.50]
Total leukocyte count (G/l)	12.27 ± 4.40	13.65 ± 4.42	0.073	+ 1.39 [-0.13 to 2.90]	13.04 ± 4.34	13.38 ± 4.74	0.618	+ 0.35 [-1.02 to 1.72]	12.67 ± 4.42	12.67 ± 4.27	0.998	-0.00 [-1.38 to 1.38]
CRP (mg/dl)	3.90 ± 5.50	6.02 ± 7.27	0.069	+ 2.12 [-0.17 to 4.41]	4.91 ± 7.46	5.91 ± 6.77	0.355	+ 1.00 [-1.13 to 3.13]	5.58 ± 7.77	4.89 ± 6.67	0.553	-0.68 [-2.96 to 1.59]
Length of stay (days)	3.28 ± 2.11	3.74 ± 1.96	0.194	+ 0.46 [-0.24 to 1.15]	3.89 ± 5.55	3.87 ± 2.70	0.975	-0.02 [-1.30 to 1.26]	3.52 ± 2.48	3.24 ± 1.80	0.413	-0.28 [-0.94 to 0.39]
Sex	male: 36 female: 52	male: 28 female: 26	0.272		male: 46 female: 48	male: 38 female: 42	0.971		male: 43 female: 56	male: 29 female: 34	0.871	
Operation technique												
0: laparoscopic	87 (98.86%)	49 (90.74%)	0.007		90 (95.74%)	73 (91.25%)	0.509		96 (96.97%)	63 (100.00%)	0.707	
1: open	1 (1.14%)	0 (0.00%)			1 (1.06%)	1 (1.25%)			2 (2.02%)	0 (0.00%)		
2: conversion	0 (0.00%)	5 (9.26%)			3 (3.19%)	6 (7.50%)			1 (1.01%)	0 (0.00%)		
Open or conversion (vs. laparoscopic)	1 (1.14%)	5 (9.26%)	0.03	+ 8.1 pp [0.9 to 18.8]	4 (4.26%)	7 (8.75%)	0.349	+ 4.5 pp [-3.1 to 13.1]	3 (3.03%)	0 (0.00%)	0.283	-3.0 pp [-8.5 to 3.1]
Postoperative antibiotics	21 (23.86%)	23 (42.59%)	0.031	+ 18.7 pp [3.0 to 34.0]	20 (21.28%)	38 (48.10%)	< 0.001	+ 26.8 pp [12.7 to 39.8]	28 (28.28%)	14 (22.22%)	0.5	-6.1 pp [-18.8 to 8.1]
Peritonitis	21 (23.86%)	23 (42.59%)	0.031	+ 18.7 pp [3.0 to 34.0]	24 (25.53%)	29 (36.25%)	0.172	+ 10.7 pp [-3.0 to 24.1]	28 (28.28%)	12 (19.05%)	0.253	-9.2 pp [-21.6 to 4.6]
Grade of appendicitis												
0: normal	7 (7.95%)	2 (3.70%)	0.001		9 (9.57%)	3 (3.75%)	0.141		7 (7.07%)	6 (9.52%)	0.433	
1: acute	71 (80.68%)	32 (59.26%)			68 (72.34%)	55 (68.75%)			72 (72.73%)	49 (77.78%)		
2: perforated/abscess	10 (11.36%)	20 (37.04%)			17 (18.09%)	22 (27.50%)			20 (20.20%)	8 (12.70%)		
Perforated/abscess (vs. other)	10 (11.36%)	20 (37.04%)	< 0.001	+ 25.7 pp [11.4 to 39.9]	17 (18.09%)	22 (27.50%)	0.193	+ 9.4 pp [-3.0 to 21.9]	20 (20.20%)	8 (12.70%)	0.309	-7.5 pp [-18.3 to 4.9]
Histologic result												
0: no appendicitis	10 (11.36%)	2 (3.70%)	0.098		11 (11.70%)	3 (3.75%)	0.078		9 (9.09%)	9 (14.29%)	0.306	
1: mild/phlegmonous	46 (52.27%)	24 (44.44%)			57 (60.64%)	46 (57.50%)			53 (53.54%)	37 (58.73%)		
2: severe/necrotic/perforated	32 (36.36%)	28 (51.85%)			26 (27.66%)	31 (38.75%)			37 (37.37%)	17 (26.98%)		
Severe histology (vs. other)	32 (36.36%)	28 (51.85%)	0.101	+ 15.5 pp [-1.2 to 31.3]	26 (27.66%)	31 (38.75%)	0.164	+ 11.1 pp [-2.8 to 24.7]	37 (37.37%)	17 (26.98%)	0.231	-10.4 pp [-24.0 to 4.6]
SARS-CoV-2 infection	0 (0.00%)	0 (0.00%)	1		0 (0.00%)	0 (0.00%)	1		0 (0.00%)	1 (1.59%)	0.389	+ 1.6 pp [-2.4 to 8.5]

Continuous variables are presented as mean ± SD and compared using Welch’s t-test. Categorical variables are reported as n (%) and compared using Chi² test or Fisher’s exact test (SARS-CoV-2 infection). For categorical variables with three categories (operative technique, grade of appendicitis, histologic result), a single global Chi² test (df = 2) was applied. Effect sizes are reported as mean differences (continuous) or absolute risk differences in percentage points (binary), with 95% confidence intervals. Dichotomized rows are provided for selected categorical variables to facilitate interpretation of effect sizes

#### Wave 3

The period from March 1 to May 31, 2021 was defined as the 3rd pandemic wave. There were 80 patients with AA identified in this period compared to 94 patients in the same period in 2019. Demographics between the two groups were similar, with no significant differences in age, sex, duration of symptoms, total leukocyte count, or CRP. Disease severity, as measured by intraoperative grade of appendicitis (*p* = 0.141), histological results (*p* = 0.078), and peritonitis rate (*p* = 0.172), did not differ significantly between groups. Length of hospital stay was comparable (3.87 vs. 3.89 days, *p* = 0.975). However, postoperative antibiotic use was significantly higher in the pandemic group (48.1% vs. 21.3%, *p* < 0.001). Overall, wave 3 showed a trend toward normalization of clinical presentation compared to wave 2, with most parameters no longer differing from pre-pandemic levels.

#### Wave 4

The period from September 1 to November 30, 2021 was defined as the 4th pandemic wave. There were 63 patients with AA identified in this period compared to 99 patients in the same period in 2019. Demographics between groups were similar. There were no significant differences in duration of symptoms, total leukocyte count, CRP, or length of hospital stay. Intraoperative and histological findings were comparable, with no significant differences in grade of appendicitis (*p* = 0.433), histological results (*p* = 0.306), peritonitis rate (*p* = 0.253), or postoperative antibiotic use (*p* = 0.500). Operative technique showed no differences, with all patients in the pandemic group undergoing successful laparoscopic appendectomy. These findings suggest that by wave 4, clinical presentation and outcomes had returned to pre-pandemic levels.

Negative appendectomy rates (histological result: no appendicitis) varied across waves. During the pandemic, rates were 3.7% (2/54) in wave 2, 3.8% (3/80) in wave 3, and 14.3% (9/63) in wave 4, compared to 11.4% (10/88), 11.7% (11/94), and 9.1% (9/99) in the corresponding pre-pandemic periods, respectively.

Throughout the study period, one patient tested positive for SARS-CoV-2 upon admission. No nosocomial SARS-CoV-2 infections were identified. 

## Discussion

### Statement of principal findings

Our study provides an overview of the changes in incidence, clinical presentation, and outcomes of acute appendicitis across three distinct COVID-19 waves over nearly two years. It indicates that the pandemic resulted in a lower number of patients with appendicitis presenting to the emergency department of our high-volume hospital compared to the pre-COVID era. In particular during periods of high SARS-CoV-2 incidence, defined as “waves”, the number of patients with AA was significantly lower.

While wave 1 was analyzed in a previous publication, the present study focuses on waves 2–4 to assess long-term patterns. The overall pandemic group (Group B), covering March 2020 to December 2021, showed no significant differences in presentation or outcomes compared to Group A (pre-pandemic). However, when looking at the individual waves, differences emerge. As in wave 1, patients during wave 2 presented with more severe disease, including significantly higher rates of perforation, peritonitis, and postoperative antibiotic use, as well as numerically elevated inflammatory markers. This finding is consistent with the results of a large number of studies and meta-analyses that analyzed the beginning of the COVID-19 pandemic [3, 12, 13, 15, 16, 18–22].

This effect was attenuated during the further course of the pandemic. In wave 3, most clinical parameters no longer differed from pre-pandemic levels, although postoperative antibiotic use remained elevated. By wave 4, presentation and outcomes were comparable to 2019. These findings suggest that the initial disruptions in emergency surgical care were transient, with presentation patterns tending toward pre-pandemic levels by wave 4.

### Interpretation within the context of the wider literature

As mentioned before, the beginning of the COVID-19 pandemic led to a decreased number of AA. Also, a reduction of elective and emergency surgery has been reported worldwide [23–26]. Meta-analyses including over 100.000 patients confirmed the reduced rate of admissions for AA and an increased proportion of complicated cases [3, 15].

Almost all studies initially focused on the beginning of the pandemic. At this time, there were strict pandemic rules in many countries. To relieve pressure on healthcare institutions, people were called upon to go to an emergency department only if necessary. More recent research now addresses longer-term effects on healthcare delivery and patient behavior, underscoring the relevance of our extended analysis. To our knowledge, this is one of the first studies that analyzes the development of incidence, clinical presentation and outcome of AA across three later pandemic waves. Our wave-based approach captures temporal patterns in disease severity, patient behavior, and health system response beyond the early shock phase. By extending the observation period into waves 2 through 4, we illustrate how emergency care delivery adapted dynamically to pandemic conditions.

The ongoing pandemic led to a rapid learning process among healthcare institutions and workers. There was not only progress in treating and preventing an infection with SARS-CoV-2, but changes were made in the workflow and organizational patterns of the hospitals. Also, strategies for emergency surgeries were implemented [27]. These changes provide important context for interpreting how care patterns for acute surgical conditions like appendicitis evolved during later pandemic waves.

A reduced number of patients presenting with AA in our emergency department was a constant finding during all high-incidence phases. The pressure especially on critical care capacity and medical staff in our center remained high especially during these waves. Local media were frequently reporting about this issue. Thus, it might be the most obvious explanation for the constantly reduced number of patients presenting with AA in our emergency department, that patients with abdominal pain avoided our institution and presented in smaller hospitals instead. Our institution normally serves not only the city of Augsburg, but also different surrounding rural areas. Patients could have preferred medical institutions closer to their residence. In accordance with this explanation, we found an increase of patients with AA in six surrounding hospitals during the first wave [14].

Still, this doesn’t fully explain the different patterns of admissions seen during the first two waves compared to the third and fourth wave and compared to the pre-pandemic era. The findings during the first two waves were similar. Fewer patients presented with AA compared to pre-COVID, but those presenting showed more advanced disease and a higher proportion of complicated appendicitis. Three reasons for the consistently described higher proportion of advanced AA are discussed: (1) There might be progression of severity because of delay. This delay can be prehospital because of “pandemic fear” or intrahospital because of limited resources [3, 14]. (2) More patients with uncomplicated appendicitis could be treated with antibiotics as an outpatient which leads to a higher proportion of severe cases in-hospital [17]. (3) Spontaneously resolving uncomplicated appendicitis may result in a higher relative number of complicated appendicitis [15]. The latter refers to resolution without any antibiotic therapy or surgical intervention.

While the first explanation should cause a higher absolute number of severe cases, the higher proportion of complicated cases is only a relative phenomenon in the second and third scenario. In our institution, histologic results did not show a consistently higher absolute number of severe cases during the four waves. This points toward the results of a recent metanalysis that demonstrated a decrease in the number of uncomplicated appendicitis and a stable incidence of complicated appendicitis [15].

In a retrospective analysis on the first and second wave of the pandemic, the authors could see a gradual return to the norm in respect to the total number of emergency surgeries despite the effects of the second wave being significantly more profound in terms of hospitalization and COVID-related mortality [28]. Similarly, in waves 3 and 4 at our institution, the proportion of complicated appendicitis returned to pre-pandemic levels. This suggests that by year two, patient behavior had changed: while tertiary centers may still have been avoided, care-seeking behavior for abdominal pain resembled pre-pandemic norms.

Vaccination against SARS-CoV-2 may also have played a role. Basic immunization started in March 2021 and by October 2021, approximately 80% of patients admitted to our hospital — across all departments — had received at least basic immunization according to institutional admission screening data, potentially reducing fear of hospital visits.

An important finding of our study is that the risk of in-hospital transmission of SARS-CoV-2 for patients with AA appeared to be very low. Based on mandatory admission PCR screening and regular repeat testing, no nosocomial infections were identified during the study period. This supports the notion that emergency surgical care could be delivered safely throughout the pandemic.

### Implications for policy, practice and research

The SARS-CoV-2 pandemic was the first “real” pandemic, that our modern health care system had to face. Despite the unpredictability of this situation, safety precautions for healthcare workers and patients made it possible to reorganize pathways in hospitals in order to treat infected patients as well as safely maintain the routine for emergency treatments. Thus, our data suggest, that during the whole pandemic, these measures were effective and safe for patients. Compared to our previous study, an extended timeframe allowed us to observe how healthcare systems and patient behavior evolved. Key findings include a trend toward pre-pandemic presentation patterns in later waves and the potential influence of the vaccination rollout. Our findings suggest that the initial disruption of emergency surgical care observed during early pandemic phases was not sustained, with presentation severity and outcomes of operated acute appendicitis tending to pre-pandemic levels in later waves.

As a lesson for future pandemic scenarios, our findings show that emergency surgical systems may be capable of adapting to sustained strain while preserving care quality. AA reflects how emergency services responded and evolved over time. Improvements such as optimized triage, selective outpatient care, shorter hospitalizations, and temporary decentralization of emergency cases were central strategies that helped hospitals maintain functionality despite high infection rates.

Importantly, patients should still be encouraged to avoid emergency departments for minor issues during pandemics. However, they must also be reassured that it remains both sensible and safe to seek urgent care in cases of serious symptoms. Our experience suggests that the initial “pandemic fear” may not recur to the same extent in future outbreaks, as both patients and healthcare providers are now better prepared. Hospitals should not assume that emergency case volumes during future pandemics will necessarily decline to the same extent as observed during the early COVID-19 waves.

Finally, vaccination likely represented one of several factors that may have supported stabilization of healthcare delivery during later pandemic phases. Further epidemiologic analyses need to be carried out in order to obtain a more comprehensive picture of the distribution of emergency cases during pandemics.

### Strengths and limitations

This study does not aim to assess population-based incidence or overall disease burden, but rather offers insights into how presentation patterns and outcomes of surgically treated acute appendicitis evolved across different phases of the COVID-19 pandemic at a tertiary referral center. A major strength is the correlation of AA case numbers and clinical outcomes with real-world SARS-CoV-2 incidence both at the institutional and regional level. Furthermore, our analysis spans a longer timeframe than most existing studies, including three distinct pandemic waves, allowing us to observe longer-term trends and behavioral adaptations.

However, this is a single-center retrospective study, which limits the generalizability of our findings. The correlation with SARS-CoV-2 incidences illustrates the impact of a pandemic on the health care system in Germany, but we cannot directly transfer these findings to other countries. Due to its retrospective design, the risk of incomplete documentation, interpretation bias or inconsistencies in the assessment of data must also be considered.

This study includes only patients who underwent appendectomy and cannot capture those managed conservatively, treated as outpatients, or never presenting to care. As non-operative management of uncomplicated appendicitis increased during the pandemic, shifts in the proportion of complicated cases may partly reflect changes in treatment strategies rather than disease severity alone.

Postoperative complications were not systematically graded according to Clavien-Dindo due to inconsistent documentation; however, surrogate markers of morbidity (peritonitis rate, antibiotic use, conversion rate, length of stay) are reported for each wave.

Notably, patients during wave 2 were significantly older than those in the corresponding pre-pandemic period (45.85 vs. 34.81 years, *p* < 0.001). As age is a known predictor of appendicitis severity and postoperative complications, this difference may have contributed to the more severe presentation observed during this wave. Given the limited sample sizes, multivariable adjustment was not performed, and the observed differences should be interpreted in the context of this potential confounder.

Our study focuses solely on acute appendicitis and does not include elective or oncologic surgeries, which may have been impacted even more by the pandemic. The retrospective design also limits our ability to assess reasons for delayed presentation or patients’ subjective experiences. Nevertheless, our histological and clinical data provide a robust basis for interpreting trends in disease severity and hospital workflow over time.

## Conclusion

This long-term, wave-based analysis indicates that the COVID-19 pandemic had a sustained impact on presentation patterns and case volume in acute appendicitis. Over the course of three pandemic waves, the number of patients presenting with acute appendicitis remained consistently lower than in the pre-pandemic period. During wave 2, patients presented with more advanced disease, including significantly higher rates of perforation and peritonitis. In wave 3, most clinical parameters no longer differed from pre-pandemic levels, although postoperative antibiotic use remained elevated. By wave 4, clinical severity and postoperative course were similar to the pre-pandemic period, despite ongoing pandemic-related challenges. These findings are consistent with the possibility that healthcare systems and patient behavior adapted over time. Acute appendicitis served as a useful tracer condition to monitor these shifts. Importantly, the extremely low rate of in-hospital SARS-CoV-2 transmission in our cohort indicates that emergency surgical care can be maintained safely even during prolonged periods of healthcare system strain. Our results emphasize the need for structured hospital workflows, flexible resource management, and clear public communication to maintain essential surgical care during future public health crises. While the retrospective and single-center design limits the generalizability of our findings, the observation that initial disruptions in emergency surgical care were not sustained across successive pandemic waves may offer reassurance that healthcare systems can adapt and recover in the face of public health crises.

## Data Availability

All data supporting the findings of this study are available within the paper.

## Abbreviations

AA: Acute appendicitis
COVID19: SARS–CoV–2 infections and coronavirus disease 2019 (COVID–19)
CRP: C–reactive protein
N: Numbers
PCR: Polymerase chain reaction
Pts: Patients
UKA: University Hospital Augsburg
